# Effect of Drain Output on the Timing of Closed Suction Drain (CSD) Removal After Head and Neck Surgery

**DOI:** 10.7759/cureus.23391

**Published:** 2022-03-22

**Authors:** Dominique Bohorquez, Stefanie Pena, Donald Weed, Ruixan Ma, David J Arnold

**Affiliations:** 1 Otolaryngology - Head and Neck Surgery, University of Miami Miller School of Medicine, Miami, USA; 2 Biostatistics, University of Miami Miller School of Medicine, Miami, USA

**Keywords:** prolonged drain use, wound complications, post-operative day, drain output, criterion, closed suction drain, head and neck surgery

## Abstract

Background: A closed suction drain (CSD) is often utilized in head and neck surgical procedures to obliterate dead space. CSDs reduce seroma and hematoma formation, thereby improving skin apposition and wound healing. The use of drains for prolonged periods of time, however, may increase the risk of wound infection. Interestingly, the evidence regarding the need for, and management of, post-operative CSDs after head and neck surgery is scarce. The current criterion of drain removal when output is less than 30 cubic centimeters (cm^3^) within a 24-hour period and/or on the third post-operative day (POD) is widely utilized. The aforementioned criterion is based on anecdotal evidence from small studies with specific surgical procedures. In this study, we aim to evaluate the criteria for drain removal and to lay the groundwork for an updated paradigm for drain management in head and neck oncologic surgery.

Study Design: Retrospective cohort study

Setting: Academic tertiary care hospital

Methods: A retrospective study was performed. Patients were included if they underwent head and neck surgery at the University of Miami Hospital between January 1, 2019 and July 1, 2020 and had at least one CSD. Volume of drain output on each POD was recorded until the day of drain removal. The development of post-operative wound complications (i.e., seroma, hematoma, infection/abscess, and dehiscence) was also recorded.

Results: From our initial cohort of 302 patients, 145 patients met inclusion criteria. A total of 10 patients developed a post-operative wound complication. Patients had a mean age of 58.3 ± 15.0 years. The median inter-quartile range (IQR) drain output (cm^3^) on the day of CSD removal from patients who developed a wound complication was similar (15; IQR, 5-37.5) when compared to those who did not develop a wound complication (25; IQR, 10-30). This difference was not statistically significant (*p* = 0.60). Additionally, the cohort who developed a post-operative wound complication had their drain removed on an earlier POD (1; IQR, 1-1 (Mean 1.2)) when compared to the cohort who did not develop any complications (1; IQR, 1-1 (Mean 1.5)). This difference was also not statistically significant (*p* = 0.48) .

Conclusion: There is no association between drain output (cm^3^) or day of CSD removal with the development of wound complications. These results warrant further studies to prospectively evaluate earlier CSD removal in head and neck surgery.

## Introduction

The use of closed suction drains (CSDs) has become a standard in the management of patients recovering from a wide range of ablative head and neck surgical procedures. The timing of removal of these drains is less well-established but has significant impact on multiple aspects of the care of these often complex patients. It is common practice to remove drains when output drops below 30 cubic centimeter (cm^3^) over a 24-hour period [1]. Allowing these criteria to be met often requires these drains to stay within the surgical bed for several days. This can lead to increased patient discomfort, increased length of stay (LOS), and increased hospital care costs. There is growing but inconclusive evidence that a more aggressive approach to drain removal is possible without raising the risk of surgical site complications. Early drain removal has multiple advantages, including but not limited to improving patient comfort and allowing earlier hospital discharges [2-5]. We have analyzed the varied practices of eight head and neck surgeons to provide clarity regarding this important aspect of post-operative care.

## Materials and methods

Institutional review board (IRB) approval at the University of Miami was obtained for this study (Protocol # 20201150). Inclusion criteria consisted of adult patients (> 18 years of age) who underwent head and neck surgery at our institution between January 1, 2019 and July 1, 2020 and had at least one CSD placed at the time of surgery. We included any patient who underwent a neck dissection, thyroidectomy (hemi or total thyroidectomy), parotidectomy (superficial, subtotal/deep lobe, or total parotidectomy), parathyroidectomy, or submandibular gland excision. Subjects were excluded if they underwent surgery in continuity with mucosal resection or if CSD removal data was incomplete (e.g., if the date of removal or output on date of removal was unknown). A total of 145 subjects operated by a single group of eight fellowship-trained head and neck surgeons met inclusion criteria. Patient demographics, clinical characteristics (e.g., history of head and neck radiation, smoking, or diabetes), type of surgery, type of wound complication (i.e., seroma, hematoma, infection/abscess, and dehiscence), daily volume of CSD output, and post-operative day (POD) of CSD removal were extracted from electronic medical records.

Statistical analysis

The demographic, clinical, and surgical characteristics were characterized with descriptive statistics, such as mean, standard deviation, median, count, and percentage. All the continuous variables were correlated with wound complication by using Two Sample T Test or Wilcoxon Rank Sum Test, as appropriate. All the categorical variables were compared with wound complication by using Fisher’s Exact Test. The significance threshold was set at p < 0.05. Statistical analyses were performed using R statistical software (version 4.0.3, R Foundation for Statistical Computing, Vienna, Austria).

## Results

Patient demographics and clinical characteristics

This study included 145 subjects who met selection criteria for inclusion and analysis. Patients had a mean age of 58.3 ± 15.0 years with a predominance of female patients (53.1%). 92.4% of patients were not active tobacco users with 42.1% of patients having a history of past tobacco use. Only 8.3% of patients had a history of head and neck radiation and 18.6% of patients had a history of diabetes mellitus.

The most common surgery type was parotidectomy (30.3%) followed by neck dissection (26.9%) and thyroidectomy (26.2%). The least common surgery was parathyroidectomy (4.1%). A total of 10 patients (6.9%) developed a post-operative wound complication. A summary of descriptive analysis of patient demographics and surgical characteristics can be found in Table 1 and Table 2, respectively. Using a Fisher's Exact Test, there was no statistically significant association between gender (*p* = 0.52), age (*p* = 0.73), current or past tobacco use (*p* = 0.56 and *p* = 0.74), history of head and neck radiation (*p* = 0.59), or history of diabetes mellitus (*p* = 0.21) and the risk of developing a post-operative wound complication. 

**Table 1:** Surgical characteristics of all patients

	All (N = 145)
Surgical Intervention Type	
Neck Dissection	39 (26.9%)
Parathyroidectomy	6 (4.1%)
Parotidectomy	44 (30.3%)
Superficial Parotidectomy	37 (25.5%)
Subtotal/Deep Lobe Parotidectomy	1 (0.7%)
Total Parotidectomy	6 (4.1%)
Submandibular Gland Excision	8 (5.5%)
Thyroidectomy	38 (26.2%)
Hemithyroidectomy	9 (6.2%)
Total Thyroidectomy	29 (20.0%)
Multiple Surgeries	10 (6.9%)
Number of Drains	
1	133 (91.7%)
2	12 (8.3)
Wound Complication	
No	135 (93.1%)
Yes	10 (6.9%)
Complication Type	
Dehiscence	2 (1.4%)
Hematoma	3 (2.1%)
Infection/Abscess	2 (1.4%)
Seroma	3 (2.1%)
None	135 (93.1%)

**Table 2:** Demographics and clinical characteristics of all patients

	All (N = 145)
Age	
Age (in years)	58.3 (mean)
Gender	
Female	77 (53%)
Male	68 (47%)
Current Tobacco Use	
No	134 (92.4%)
Yes	11 (7.6%)
Past Tobacco Use	
No	84 (57.9%)
Yes	61 (42.1%)
History of Head and Neck Radiation	
No	133 (91.7%)
Yes	12 (8.3%)
History of Diabetes Mellitus	
No	118 (81.4%)
Yes	27 (8.6%)

Drain output volume on the day of drain removal

**Figure 1:**
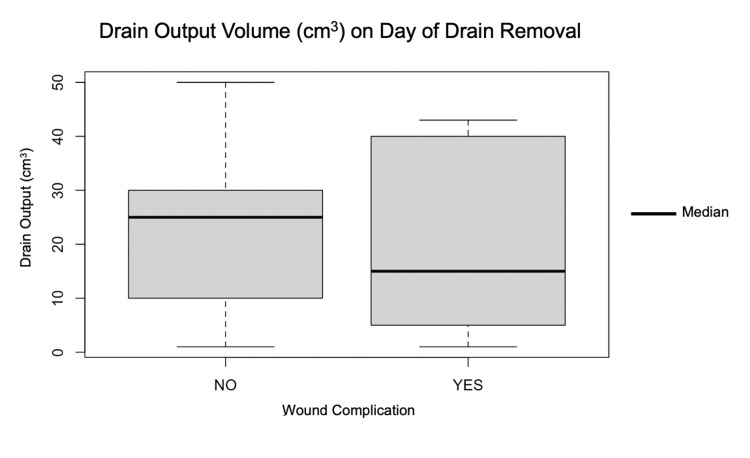
Drain output volume (cm3) on the day of drain removal

The median inter-quartile range (IQR) drain output (cm^3^) on the day of CSD removal from patients who developed a wound complication was similar (15; IQR, 5-37.5) when compared to those who did not develop a wound complication (25; IQR, 10-30). This difference was not statistically significant (*p* = 0.60) (Figure 1).

POD of drain removal

**Figure 2:**
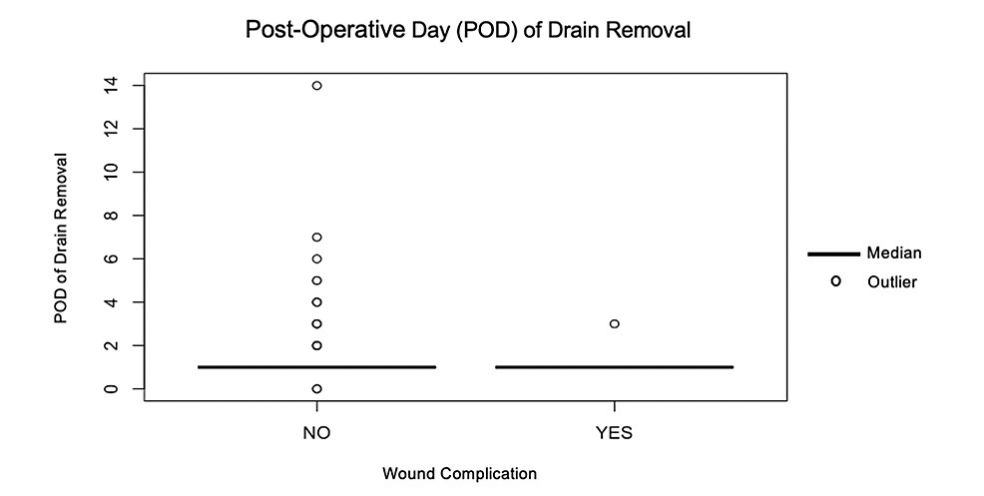
Post-operative day (POD) of drain removal

The cohort who developed a post-operative wound complication had their drain removed on an earlier post-operative day (1; IQR, 1-1 (Mean 1.2)) when compared to the cohort who did not develop any complications (1; IQR, 1-1 (Mean 1.5)). This difference was not statistically significant (*p* = 0.48) (Figure 2).

## Discussion

CSDs have been widely used by head and neck surgeons worldwide to evacuate blood and exudate in the early post-operative period. It is felt that this practice promotes skin flap healing by avoiding hematoma or seroma formation. This is aided by active suction, which physically opposes skin flaps to the underlying wound bed allowing for wound healing. Based on our experience supporting these beliefs, it has become routine for drains to be left in place for multiple days in the post-operative period [1]. There is sporadic but growing data to support the idea that drains can be discontinued earlier than is current practice without causing adverse effect on wound healing. Analysis of our current experience supports this notion.

The ability to remove drains earlier can improve patient care in several ways. Drains cause significant site discomfort for patients and require the creation of an additional scar. They can also contribute to post-operative complications linked to decreased mobility (e.g., venous stasis) [6]. Drain placement increases the risk of surgical site infection which has been shown to increase length of hospital stay, associated cost of care, and mortality [7]. 2-5% surgical patients develop wound infections with head and neck oncologic patients having an even higher rate of surgical site infections, with studies reporting the incidence as high as 87% [6]. Martone et al. estimated that 0.64% of hospital deaths are related to surgical site infections [8].

As these risks and their association with CSD have become clearer, there has been an increased interest in minimizing or avoiding the use of drains altogether, especially in cases where the risk of doing so seemed minimal. Thyroid surgery has provided an obvious opportunity to decrease the use of CSD and there has been significant work showing that thyroidectomy may be performed without placing a drain in many cases [2,9]. It has been postulated that the very presence of a drain may induce output by causing inflammation and by preventing open lymphatics from self-sealing as a result of suction in the field [7]. It has also been argued that the drain itself may act as a foreign body, stimulating the formation of seroma and creating a potential space for fluid accumulation [3].

To date, a number of studies have focused on post-operative drain management in specific populations of post-surgical patients [3,4,10]. However, few studies have focused on head and neck surgery [5]. It is therefore not surprising that there is no clear consensus regarding the management of post-operative drains in head and neck cancer patients. Panda et al. studied a group of 153 patients and found that most surgical site drainage occurred within the first 24 hours of surgery. They concluded that it was safe to remove drains 48 hours after major head and neck surgery [5]. They also noted that the quantity of drainage was not associated with the type of procedure performed. Harris et al. studied 47 patients undergoing major head and neck surgery and concluded that drains can be removed when output dropped below 50 cm^3^ in a 24-hour period [11].

We found no association between drain output or day of CSD removal and the development of post-operative wound complications and have provided a more comprehensive look at the use of CSDs. We have demonstrated that there is no association between the timing of drain removal or quantity of drain output and wound complication in our survey of 145 patients undergoing a range of ablative head and neck surgeries.

Surgical cases in continuity with mucosal resection were specifically excluded from this analysis as the goal of this study was to evaluate and determine the conditions under which a post-operative wound could heal without the need for continued surgical drainage. The potential addition of fistula output to fluid burden was felt to likely complicate this analysis but does warrant analysis in a future prospective study.

Limitations of this work include inherent disadvantages associated with retrospective studies such as missing and incomplete patient information. Moreover, as a result of the low frequency of wound complications associated with CSD, we were only able to gather a small cohort of patients who experienced a CSD-associated wound complication. Additionally, confounding variables such as history of head and neck radiation and clinical history of diabetes mellitus were not controlled for by multivariate analysis because insufficient frequency did not allow statistical significance. Instead, a Fisher's Exact Test was performed which demonstrated no statistically significant association between history of diabetes mellitus or previous radiation and development of wound complication. Future directions include conducting a prospective randomized clinical trial where we control for confounding variables with the goal of establishing an updated protocol for CSD management following head and neck surgery.

## Conclusions

There is no clear association between wound complication and the timing of drain removal or drain output on the day of removal in the treatment of complex post-operative head and neck disease in our series of 145 patients. The potential benefits of improving patient comfort and minimizing hospital LOS warrant a careful prospective study with the goal of improving outcomes and efficiency in the management of head and neck post-surgical patients.
